# Integrating animal experiments, mass spectrometry and network-based approach to reveal the sleep-improving effects of Ziziphi Spinosae Semen and γ-aminobutyric acid mixture

**DOI:** 10.1186/s13020-023-00814-9

**Published:** 2023-08-12

**Authors:** Airong Ren, Tingbiao Wu, Yarong Wang, Qing Fan, Zhenhao Yang, Shixun Zhang, Yongjun Cao, Guozhen Cui

**Affiliations:** 1 Department of Bioengineering, Zhuhai Campus of Zunyi Medical University, Zhuhai, 519000 Guangdong China; 2 Basic Medical Science Department, Zhuhai Campus of Zunyi Medical University, Zhuhai, 519000 Guangdong China

**Keywords:** Ziziphi Spinosae Semen, γ-aminobutyric acid, Sleep improvement, Mass spectrometry, Network medicine, Proximity, Quality markers

## Abstract

**Background:**

Ziziphi Spinosae Semen (ZSS) is a plant widely used as medicine and food in Asian countries due to its numerous health benefits. γ-aminobutyric acid (GABA), a non-proteinaceous amino acid, is one of the major inhibitory neurotransmitters with a relaxant function. In this study, a system pharmacology approach was employed to assess the effects of a mixture composed of ZSS and GABA (ZSSG) on sleep improvement.

**Methods:**

Mice were divided into five groups (n = 10) and received either no treatment, sodium pentobarbital, or sodium barbital with diazepam or ZSSG. The effects of ZSSG on sleep quality were evaluated in mice, and differential metabolites associated with sleep were identified among the control, ZSS, GABA, and ZSSG groups. Additionally, network-based ingredient-insomnia proximity analysis was applied to explore the major ingredients.

**Results:**

ZSSG significantly improved sleep quality by decreasing sleep latency and prolonging sleep duration in sodium pentobarbital-induced sleeping mouse model (*P* < 0.05). ZSSG significantly enhanced the brain content of GABA in mice. Furthermore, ZSSG also significantly decreased sleep latency-induced by sodium barbital in mice (*P* < 0.05). Metabolic analysis revealed significant differences in 10 metabolites between ZSSG group and the groups administering ZSS or GABA. Lastly, using the network-based ingredient screening model, we discovered potential four active ingredients and three pairwise ingredient combinations with synergistic effect on insomnia from ZSSG among 85 ingredients identified by UPLC-Q/TOF–MS. Also, we have constructed an online computation platform.

**Conclusion:**

Our data demonstrated that ZSSG improved the sleeping quality of mice and helped to balance metabolic disorders-associated with sleep disorders. Moreover, based on the network-based prediction method, the four potential active ingredients in ZSSG could serve as quality markers-associated with insomnia. The network-based framework may open up a new avenue for the discovery of active ingredients of herbal medicine for treating complex chronic diseases or symptoms, such as insomnia.

**Supplementary Information:**

The online version contains supplementary material available at 10.1186/s13020-023-00814-9.

## Introduction

Insomnia is a prevalent mental disorder characterized by difficulty falling asleep, short overall sleep duration, and frequent awakenings, which seriously affects the physical and mental health of people, leading to reduced quality of life in persons who suffer from insomnia. Approximately 33%-50% of people worldwide have experienced insomnia, especially since the outbreak of Corona Virus Disease 2019 (COVID-19), leading to a significant increase in the number of individuals afflicted by insomnia [1]. The causes of insomnia are not fully understood but are likely to involve multiple factors, including anxiety, depression, and physical discomfort. In clinical practice, pharmacological treatments, such as benzodiazepine agonists and antidepressants, are commonly used [2]. The main pharmacological mechanisms of anti-insomnia are the inhibition of wake-promoting neurotransmission and the enhancement of the inhibitory neurotransmitter GABA [3]. However, these treatments, such as oral benzodiazepine hypnotics, exhibit certain unwanted side effects including tolerance, lethargy, and addiction [4]. In contrast, traditional Chinese herbal medicines gained increasing attention in the treatment of insomnia due to their relative safety, long history, and multi-component and multi-target characteristics [5].

The mature seed of Ziziphi Spinosae Semen (ZSS), known as Suanzaoren in China, is considered a medicine food homology plant promulgated by the Ministry of Health of China. It has been well accepted that ZSS exhibits various beneficial effects, including anti-aging, anti-inflammatory, and anti-oxidant effects. In particular, as a food supplement, it is widely used for the management of insomnia and palpitations in traditional Chinese medicine [6, 7]. Currently, although it was reported that ingredients from ZSS conferred homeostatic sleep function as GABA neurotransmitter agonists in vitro and in vivo, its pharmacological profile remains to be fully characterized [6, 8].

γ-aminobutyric acid (GABA) is a major inhibitory neurotransmitter in the central nervous system and plays an important role in the regulation of neuronal excitability [9]. Usually, GABA is present in approximately 25%-50% of neurons and is a major inhibitory neurotransmitter in humans. GABA is a four-carbon non-protein amino acid found mainly in foods, such as tea, fermented milk, wheat germ, and fermented meat products, and exhibits a variety of physiological functions such as improving sleep quality and promoting relaxation [10]. It was noted that GABA has been listed as a new food additive in China since 2009. Currently, GABA has been extensively used in pharmaceuticals and functional foods.

In this study, ZSS aqueous extract and GABA (ZSSG) were combined in a certain proportion (mass ratio of raw ZSS and GABA: 6/1) to explore the effects of the mixture on promoting sleep quality in mice, and then metabolic differences between the groups receiving individual substances and the combined treatment were compared. Subsequently, the network medicine framework was used to analyze the main active components of ZSSG in the treatment of insomnia and the potential anti-insomnia mechanism. Our results will provide theoretical and experimental support for the development of sleep-improving health food.

## Materials and methods

### Reagents and preparation of ZSSG extract

Dry ZSS was obtained from Hebei Huadu Pharmaceutical Co., Ltd (Hebei, China) and GABA was obtained from Shanghai Toong Yeuan Food Tech Co., Ltd (Shanghai, China). Diazepam (Diaz) was purchased from Shandong Xinyi Pharmaceutical Co., Ltd (Shandong, China). Sodium pentobarbital and sodium barbital were obtained from Sigma-Aldrich (St. Louis, MO, USA). All reagents and solvents were commercially available as analytical grade.

Dry ZSS was washed and then pulverized after drying. The powders were screened through 6-mm mesh, distilled water was added, and the mass concentration of crude ZSSG was set at 1.55 g/mL. The mixture was refluxed for 1 h and this process was repeated three times. Subsequently, a high concentration ZSS extract was prepared by a rotary evaporator (vacuum − 0.08 to − 0.10 MPa) at 50–70 °C and then dried under reduced pressure (− 0.08 to − 0.10 MPa, 50–70 °C). After preliminary animal experimental exploration, the dry powder of the ZSS extract and GABA was weighed and mixed in a 63:50 ratio (equivalent to mass ratio of raw ZSS and GABA: 6/1). Finally, the solution was filtered through a 0.22 membrane and the compounds of ZSSG were identified by ultra-performance liquid chromatography-quadrupole time of flight-tandem mass spectrometry (UPLC-Q/TOF–MS) analysis.

### Animals and treatment

Specific pathogen-free (SPF) grade male BALB/c mice weighing 18–20 g were obtained from Guangdong Medical Laboratory Animal Centre. Mice were maintained under a 12 h/12 h light/dark cycle at ambient temperature (22–24 °C) and constant humidity (30–50%) with free access to food and water. All animals were cared for according to institutional guidelines for animal care, animal experiments were approved by the Ethics Committee for Animal Experiments of Zunyi Medical University.

The assessment methods for evaluating sleep quality improvement were performed based on the Technical Standards for Testing and Assessment of Health Food guidelines. For sodium pentobarbital and sodium barbital-induced sleep tests, the mice were randomly divided into 5 groups: control group (Ctrl), sodium pentobarbital or sodium barbital group (Pent or Barb), diazepam (Diaz, 2.50 mg/kg), and different doses of ZSSG (0.19, 0.57 g/kg). The tested samples were administered by oral gavage daily for 30 days, n = 10 mice/group. Mice in the remaining two groups were treated with an equal volume of distilled water once daily by oral gavage for 30 consecutive days. The high dose (0.57 g/kg) in mice is approximately 30 times the recommended daily human dose based on body surface area.

### Direct sleep test in mice

The sleep and wake states of the mice were observed after administration of 2 doses of ZSSG for 30 days, while the control group was given the same volume of water. Sleep was indicated by the disappearance of righting themselves from a supine to a prone position in mice. In detail, when the mice were placed in the dorsal recumbent position, if they could not turn over for more than 30–60 s, it was considered that the reflex had disappeared, indicating that the mice had fallen asleep. The animals were considered to be awake when the reflex was restored, and the time between the disappearance of the reflex and its restoration was considered sleep duration.

### Test of the prolonged duration of sodium pentobarbital-induced sleeping mouse model

Based on sodium pentobarbital hypnosis, if the sleep duration was prolonged, then the tested samples had a synergistic effect with sodium pentobarbital. First, pre-experiments were performed to determine the dose of sodium pentobarbital (54 mg/kg) that would put the animals to sleep 100% of the time, and formal experiments were performed with this dose. After the mice were administered the solvent and different doses of ZSSG, freshly prepared sodium pentobarbital was intraperitoneally administered to each group of animals. The disappearance of the righting reflex was used as an indicator to observe whether the tested samples could prolong the sleep duration of sodium pentobarbital.

### Assay for the brain content of GABA in sodium pentobarbital-treated mice

Brain samples were collected as previously described [11]. Briefly, the brain samples were homogenized with ice-cold sodium chloride solution (w/v, 1:9). The homogenate was centrifuged at 4,000 g for 15 min at 4 °C. The supernatant was collected for further analysis. The content of brain GABA was measured using a commercial ELISA kit (Senbejia Biotechnology Co., Ltd, Nanjing, China) according to the manufacturer’s instructions.

### Test on the decreased sleep latency of sodium barbital-induced sleep in mice

Based on sodium barbital hypnosis, it was observed whether ZSSG could shorten sleep latency (the time to fall asleep), and if sleep latency was shortened, then ZSSG had a synergistic effect with sodium barbital. Pre-experiments were performed to determine the dose of sodium barbital (270 mg/kg) that allowed all animals to sleep, and formal experiments were performed with this dose. After the animals were last administered the solvent and different concentrations of the tested samples for 30 min, each group of animals was injected intraperitoneally with freshly prepared sodium barbital. The sleep latency of each mouse was recorded.

### Animal treatment and collection of brain samples for metabolic analysis

To explore the different metabolites of the ZSS, GABA and ZSSG on sleep regulation, we conducted a study on BALB/c male mice. The mice were randomly divided into four groups (6 mice for each group): control group, ZSS group (0.32 g/kg), GABA group (0.25 g/kg), and ZSSG group (0.57 g/kg). After 30 min of treatment, the mice were sacrificed, and brain samples were collected. Subsequently, brain samples (0.2 g) were added to 2 mL methanol and then homogenized for 30 s. The mixtures were centrifuged for 10 min at 14 000 g at 4 °C. The supernatant was transferred into a new Eppendorf tube and then evaporated to dryness by nitrogen stream. The dry extract was then reconstituted in 1 mL methanol, and centrifuged for 10 min at 14 000 g at 4 °C. The supernatant was then filtered and subjected to LC–MS analysis [12].

### UPLC-Q/TOF–MS/MS analysis

We used a Waters Acquity UPLC I-Class system for the chromatographic separations. Separations were performed on an Acquity UPLC HSS T3 Column (2.1 × 100 mm, 1.8 μm) maintained at 40 °C with a flow rate of 0.3 mL/min and an injection volume of 1 μL after equilibration. A 0.1% formic acid (FA) aqueous solution (solvent A) and acetonitrile (solvent B) were employed as the mobile phases. The gradient profile for ZSSG analysis was set as follows: 0–2 min, 10% B; 2–16 min, 10%-90% B; 16–17 min, 90% B; 17–18 min, 90%-10% B; 18–20 min, 10% B. An increasing gradient of solvent B (v/v) was applied for metabolite analysis, as follows: 0–2 min, 2% B; 2–12 min, 2%-100% B; 12–15 min, 100% B; 15–17 min, 100%-2% B; 17–18 min, 2% B.

Mass spectrometric detection was conducted on a Waters SYNAPT XS Q-TOF mass spectrometer (Waters Corporation, Milford, USA) equipped with an electrospray ionization (ESI) source. Full scan data were acquired in the range of 50–1200 Da, using a capillary voltage of 3.0 kV for positive ion mode and -2.5 kV for negative ion mode. The cone voltage was set at 20 V, and the source temperature was maintained at 110 °C. Additionally, a cone gas flow of 50 L/h, a desolvation gas (N2) flow of 800 L/h, and a desolvation gas temperature of 350 °C were employed. The collision voltage was set at 6.0 eV for low-energy scans and 20–80 eV for high-energy scans. Data were centroided and mass-corrected during acquisition using an external reference (LockSpray™), consisting of a 100 pg/mL solution of leucine-enkephalin infused at a flow rate of 15 μL/min via a lockspray interface. This generated a real-time reference ion of [M + H]^+^ (m/z 556.2771) in positive ion mode and [M-H]^−^ (m/z 554.2615) in negative ion mode, ensuring accurate MS analysis. All data collected in centroid mode were used to calculate the accurate mass and composition of relative target ions with MassLynx™ V4.1 software (Waters Corporation, Milford, USA).

### The establishment of a chemical library for ZSSG and its metabolites

Compounds of ZSSG and metabolites were collected by searching databases such as the China Journals of Full-text Database (CNKI), Medline, PubMed, Web of Science, and ChemSpider. A custom library of chemical compounds was established using UNIFI software, incorporating compound names, molecular formulas, chemical structures, and accurate molecular masses. All MS data analysis was conducted on the UNIFI software platform. A minimum peak area of 100 was set for 2D peak detection. Selected parameters for 3D peak detection included peak intensities of high energy exceeding 20 counts and low energy exceeding 40 counts. A margin of error of up to 10 ppm for identified compounds was permitted, and matching compounds were generated with predicted fragments derived from their structures. We selected positive adducts, including H^+^/Na^+^, and negative adducts containing HCOO^−^ and H^−^. Cross adduct combinations were allowed.

### Protein target collection of ZSSG ingredients

To collect the protein targets of identified ingredients from ZSSG, we assembled the data of physical ingredient-target interactions from 6 database sources: BindingDB [13], comparative toxicogenomics database (CTD) [14], DrugCentral [15], HIT 2.0 [16], IUPHAR Guide to IMMUNOPHARMACOLOGY [17], therapeutic Target Database (TTD) [18] and ITCM database [19]. For the network analysis, we only considered the protein targets that had experimental evidence curated from the literature.

### Construction of the human protein–protein interactome

The human interactome was constructed according to 18 databases and the literature as described [20–23]. High-quality human protein–protein interactions (PPIs) from experiments were assembled. The genes were transformed into Entrez IDs according to the National Center for Biotechnology Information (NCBI) database. A comprehensive list of 485,385 unique human PPIs connecting 18,375 proteins (nodes) with a largest connected component (LCC) was constructed.

### Manual curation of human insomnia-associated genes

In the current study, we collected disease-associated genes for insomnia (MeSH Unique ID: D007319) defined by Medical Subject Headings (MeSH) and Unified Medical Language System (UMLS) [24]. We integrated disease-associated gene annotation data from different data sources: DrugBank [25], PharmGKB [26], TTD [18], and UniProt [24]. The genes were trimmed for quality assurance and only the genes that have been shown to have a direct relationship in gene knock-out mice were retained. After these steps, insomnia-related genes were also collected by manual literature searching. To ensure the completeness of insomnia-associated genes, we also chose to retrieve entries containing the synonyms “sleeplessness”, “insomnia”, “insomnia disorders”, “psychophysiological insomnia” and “disorders of initiating and maintaining sleep”. The items were converted into the corresponding Entrez IDs through the NCBI database and saved in CSV format for subsequent data processing.

### Calculation of network proximity

The network proximity between insomnia disease and an ingredient from ZSSG was calculated using a closest distance measure that reflected the path lengths between disease genes and ingredient protein targets. Here, S represents insomnia-associated genes, T represents ingredient targets and ${d}_{c}\left(S,T\right)$ represents the closest distance between S and T in the human interactome. We calculated the network proximity of S and T via Eq. (1) according to a previously described method [20].

1 $${d}_{c}\left(S,T\right)=\frac{1}{\Vert T\Vert }\sum_{t\in T}\underset{s\in S}{\mathrm{min}}d\left(s,t\right)$$

To evaluate the statistical significance of the network distance between an ingredient and insomnia, a reference distance distribution was produced by calculating the proximity between two randomly selected groups and repeated 1000 times. The mean distance ${\mu }_{{d}_{c}}\left(S,T\right)$ and standard deviation ${\sigma }_{{d}_{c}}\left(S,T\right)$ of the reference distribution were used to calculate ${Z}_{{d}_{c}}$ (Z-score) via Eq. (2). The *P*-value was also calculated using the reference distribution by counting the number of proximities that were less than the corresponding ${d}_{c}\left(S,T\right)$ and dividing the number by 1000. *Z*-scores < 0 and *P* < 0.05 were considered to be significant as previously reported [20, 21].

2 $${Z}_{{d}_{c}}=\frac{d\left(S,T\right)-{\mu }_{{d}_{c}}\left(S,T\right)}{{\sigma }_{{d}_{c}}\left(S,T\right)}$$

### Network-based rational prediction of ingredient combinations

To identify potential ingredient combinations, the top ingredients ranked by *Z*-score were combined, and their network-based relationship between ingredient targets and insomnia-associated proteins (ingredient-ingredient-insomnia combinations) was quantified. Subsequently, S_AB_ was calculated for each pair of ingredients A and B using Eq. (3) as described previously [27, 28].

3 $${S}_{AB}\equiv \langle {d}_{AB}\rangle -\frac{\langle {d}_{AA}\rangle +\langle {d}_{BB}\rangle }{2}$$

${S}_{AB}$ represents the degree of separation between the two target sets of ingredients A and B. For ${S}_{AB} < 0$, the targets of the two ingredients are in the same network neighborhood, while for ${S}_{AB} \ge 0$, the targets are topologically separated. In the calculation of ${S}_{AB}$, $\langle {d}_{AB}\rangle$ is the average shortest distance between A-B target pairs similar to the calculation of ${d}_{c}\left(S,T\right)$ via Eq. (1) by replacing the “min” function with the “mean” function. $\langle {d}_{AA}\rangle$ and $\langle {d}_{BB}\rangle$ measure the averaged distance of the target sets to each other of the same ingredient, similar to the calculation of $\langle {d}_{AB}\rangle$ with the exception that the distance between the same target (distances of a target to itself) is ignored. The “Complementary Exposure” pattern (Z_CA_ < 0, Z_CB_ < 0, *P* < 0.05, ${S}_{AB} \ge 0$) was used to predict the efficacious ingredient combinations as previously described [21, 28]. In addition, to accommodate the needs of a broader range of researchers, we have developed and launched a dedicated online calculation platform. This platform allows users to submit tasks related to network proximity calculation and provides free access to the computational results (http://www.zmupredict.cn/proximity).

### Network diagram construction

*Z*-score of the ingredient-disease was obtained through the calculation of the network proximity. After the direct physical connections between all ingredients and disease were calculated, the corresponding targets (proteins) of ingredients and disease in the PPI were extracted (http://www.zmupredict.cn/sub_net). These extracted nodes were then imported into Gephi 0.9.2 software (https://gephi.org) for network visualization analysis.

### Statistical analysis

Data were statistically analyzed using GraphPad Prism 8.0, and the results are expressed as the mean ± SD. The comparisons between multiple groups were carried out by one-way ANOVA followed by Tukey’s multiple comparisons test. For comparison between the two groups, a two-tailed t-test was used. A value of *P* ≤ 0.05 was considered statistically significant.

## Results

### Direct effect of ZSSG on sleep in mice

**Fig. 1:**
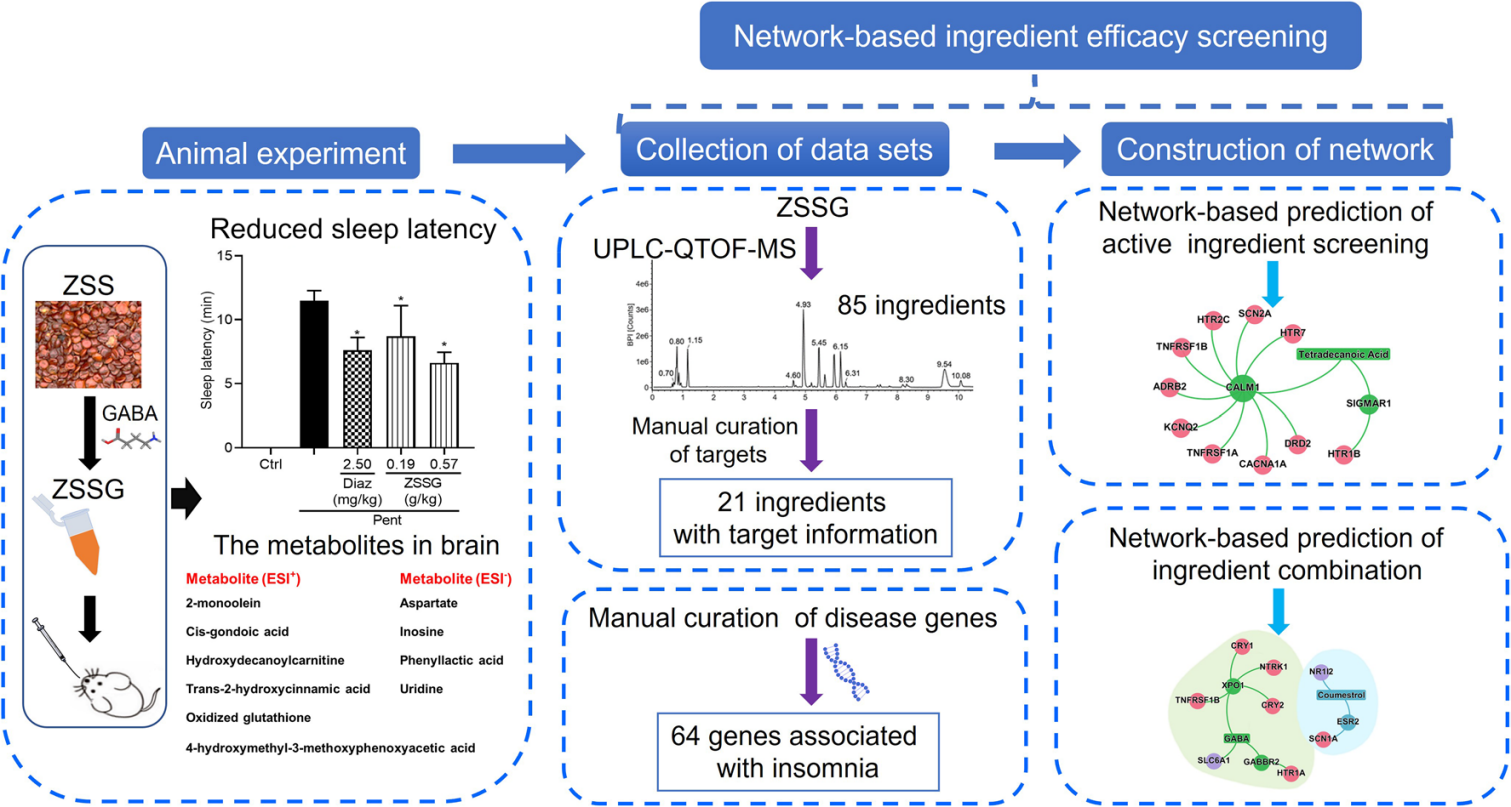
A diagram illustrating the network-based active ingredient efficacy screening for insomnia

In order to investigate the effects of ZSSG on improving sleep quality and to explore its pharmacodynamic material foundation, we have conducted systematic experimental research (Fig. 1). First, the sleep status of each mouse administered ZSSG (0.19, 0.57 g/kg) was measured. The mice were observed for 30 min after receiving the last oral gavage with the corresponding tested samples. The experimental results showed that in all experimental mice, the disappearance of righting themselves from a supine to a prone position was not observed (data not shown). These results indicate that ZSSG did not directly promote sleep in mice.

### Effects of ZSSG on sleep latency and duration in sodium pentobarbital-induced sleeping mouse model

**Fig. 2:**
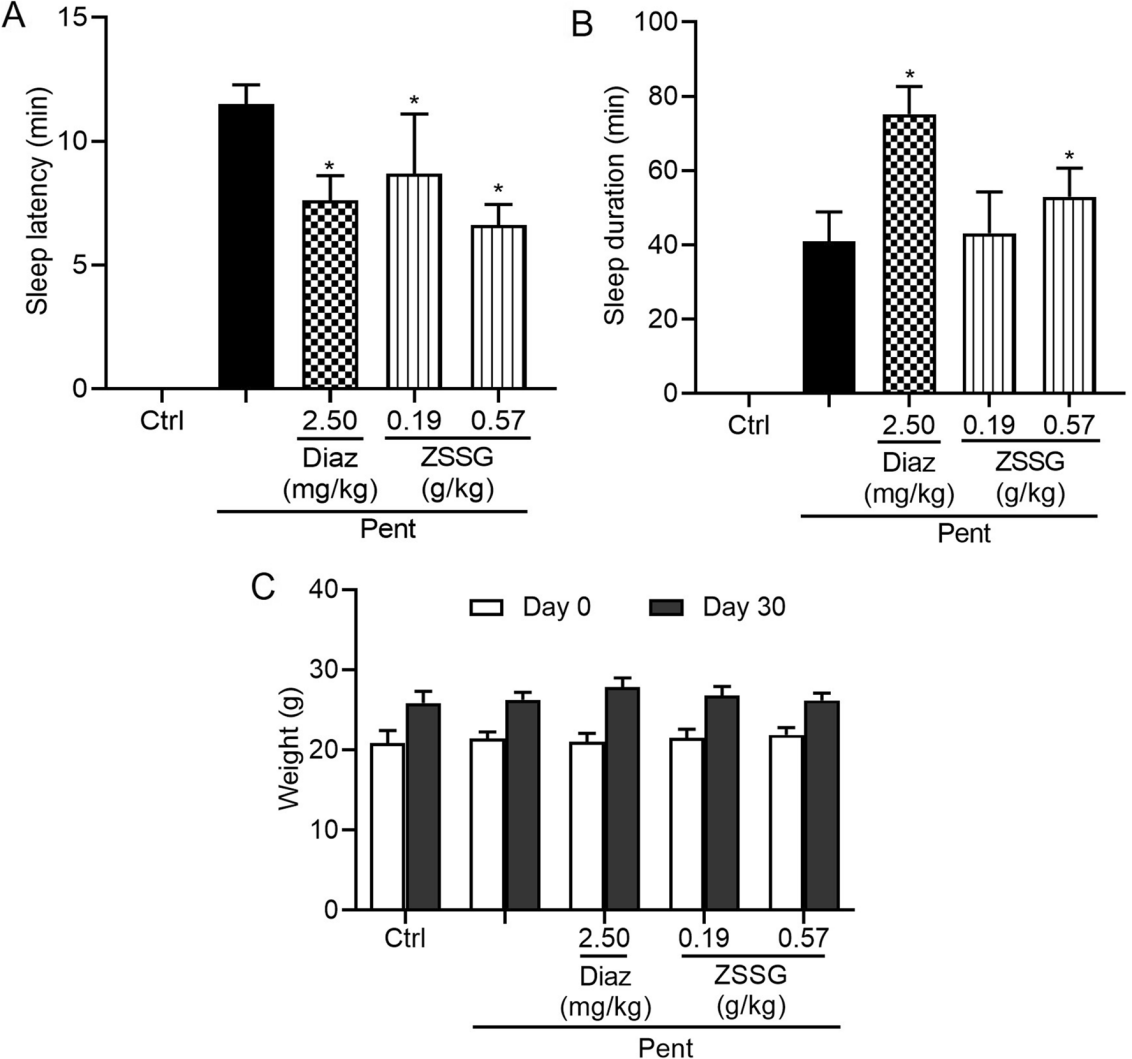
Effects of the oral administration of ZSSG in sodium pentobarbital treated mice. Mice were orally administered drinking water, Diaz (2.50 mg/kg), or ZSSG (0.19, 0.57 g/kg) for 30 consecutive days. Sodium pentobarbital (54 mg/kg)-induced sleep tests were carried out. The sleep latency (**A**), sleep time (**B**), and body weight of the mice were recorded (**C**). Each column represents the mean ± SD (n = 10), *P < 0.05 compared with the vehicle group. *Ctrl* control; Diaz: diazepam, *ZSSG* Ziziphi Spinosae Semen aqueous extract and GABA

Next, the effects of ZSSG on sodium pentobarbital (54 mg/kg)-induced sleep were evaluated in mice. As shown in Fig. 2A, Diaz (2.50 mg/kg) and ZSSG (0.19, 0.57 g/kg) significantly shorten sleep latency in mice (*P* < 0.05) compared with sodium pentobarbital group. In contrast, Diaz (2.50 mg/kg) and ZSSG (0.57 g/kg) significantly prolonged sleep duration in mice (Fig. 2B, *P* < 0.05). The initial and final body weights of the animals in the various groups are presented in Fig. 2C. Compared with sodium pentobarbital group, no significant difference in body weight was found between Diaz and ZSSG treated groups at the beginning (day 0) or the end (day 30) of the experiment. These data indicate that ZSSG can prolong sleep duration in sodium pentobarbital-treated mice.

### Effects of ZSSG on the brain content of GABA in mice

**Fig. 3:**
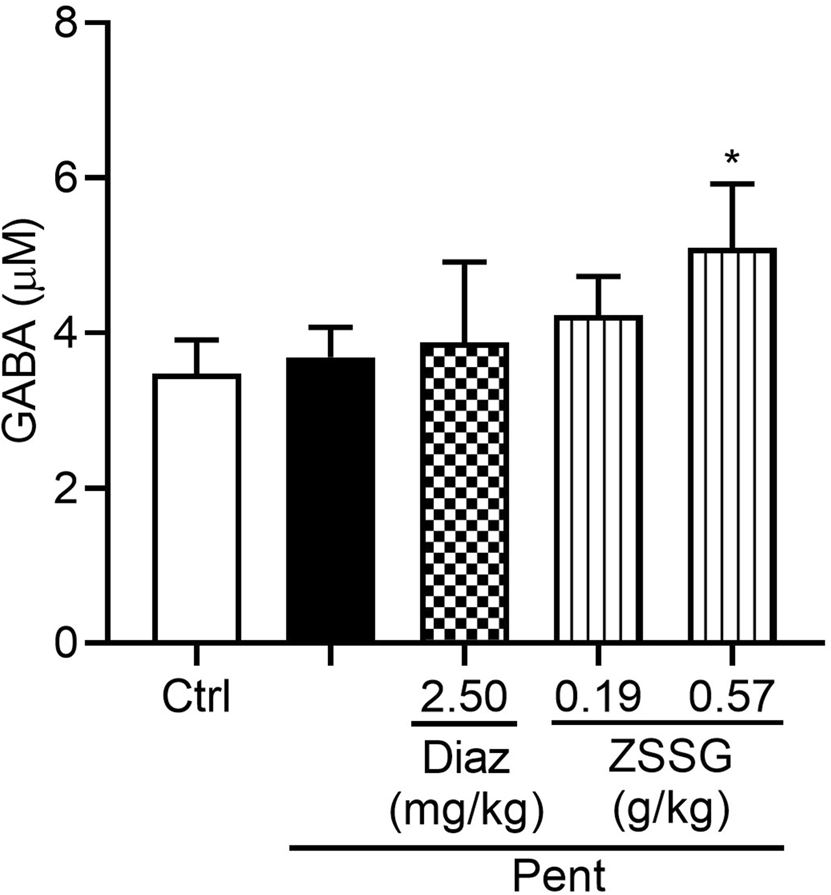
Effects of ZSSG on the brain content of GABA in sodium pentobarbital treated mice. Each column represents the mean ± SD (n = 10), *P < 0.05 compared with the vehicle group. Ctrl: control

GABA is an important inhibitory neurotransmitter in the central nervous system and favors sleep by activating GABA receptors [29]. As shown in Fig. 3, compared with sodium pentobarbital group, ZSSG at a high dose (0.57 g/kg) significantly increased the brain content of GABA in mice (*P* < 0.05).

### Effects of ZSSG on sleep latency in sodium barbital-induced sleeping mouse model

**Fig. 4:**
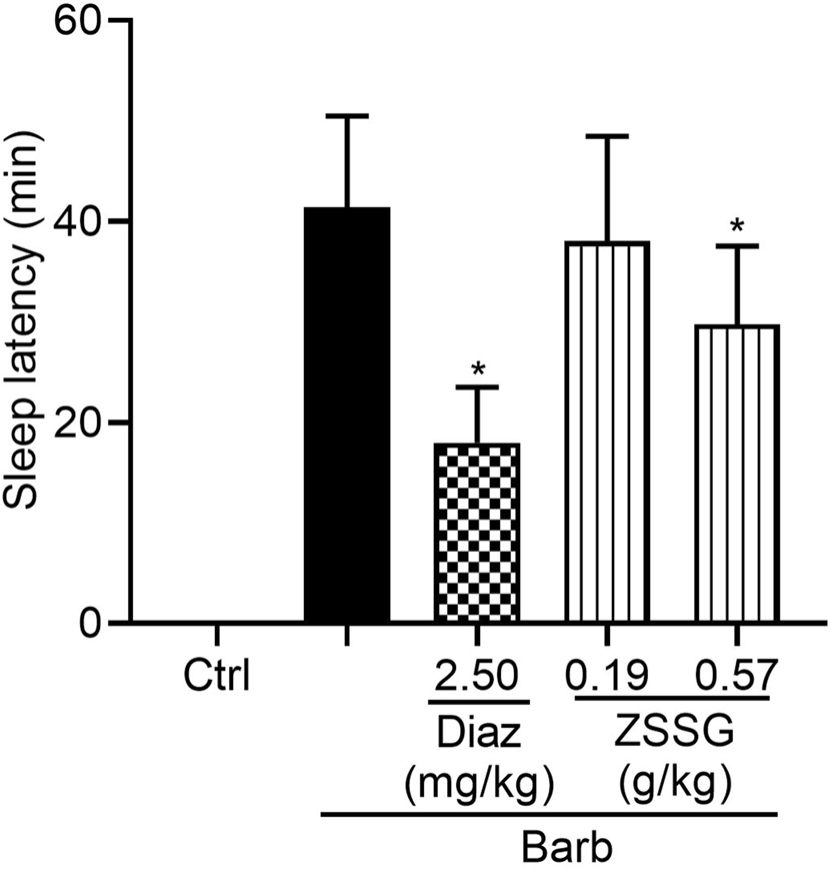
Effects of the oral administration of ZSSG on sodium barbital-induced sleep in mice. Mice were orally administered drinking water, Diaz (2.50 mg/kg) or ZSSG (0.19, 0.57 g/kg) for 30 consecutive days. Sodium barbital (270 mg/kg)-induced sleep tests were carried out and the sleep latency of the mice was recorded. Each column represents the mean ± SD (n = 10), *P < 0.05 compared with the vehicle group

The sleep latency of sodium barbital-treated mice administered Diaz and ZSSG was measured. Compared with sodium barbital group, Diaz (2.50 mg/kg) and ZSSG (0.57 g/kg) significantly decreased sleep latency (Fig. 4, *P* < 0.05). Once again, these data indicate that ZSSG can improve sleep quality in sodium barbital-treated mice.

### The different metabolites among control, ZSS, GABA and ZSSG groups

To determine whether ZSSG might have advantages in regulating the metabolic pattern-associated with sleep compared with the single-agent ZSS or GABA, we used UPLC-Q/TOF–MS for comprehensive analysis of metabolites among the control, ZSS, GABA and ZSSG groups. Differential metabolites were defined as variables with *P* values of *P* < 0.05, which allowed us to distinguish between the two groups. Compared with the single-agent (ZSS or GABA) treatment group, metabolic analysis revealed that 10 metabolites had significant changes (*P* < 0.05) in mouse brain tissue following ZSSG treatment. Specifically, 4 metabolites changed in the negative mode, while 6 changed in the positive mode. Table 1 displays the changing trends of metabolites between groups, which showed that the levels of uridine, 4-hydroxymethyl-3-methoxyphenoxyacetic acid, cis-gondoic acid, hydroxydecanoyl carnitine, and trans-2-hydroxycinnamic acid were decreased. In contrast, the level of inosine was increased compared to the single-agent treatment group. These findings suggest that these metabolites may be responsible for the ZSSG-induced effects of improving sleep in mice. Therefore, the identified metabolites can be considered as potential biomarkers for assessing the efficacy of ZSSG in treating sleep-related disorders.

**Table 1:** Differential potential metabolites characterized in mice brains and change trends

Mode	No	Metabolite	Formula	m/z	RT (min)	ZSSG vs ZSS			ZSSG vs GABA	
						Fold change	Trend		Fold change	Trend
ESI^−^	1	Aspartate	C_4_H_7_NO_4_	132.03	0.43	0.79	↓*		0.82	↓
	2	Inosine	C_10_H_12_N_4_O_5_	267.07	0.46	1.13	↑*		1.13	↑*
	3	Phenyllactic acid	C_9_H_10_O_3_	165.05	4.67	0.69	↓		0.49	↓*
	4	Uridine	C_9_H_12_N_2_O_6_	289.07	7.29	0.70	↓*		0.68	↓*
ESI^+^	5 6	2-monoolein 4-hydroxymethyl-3-methoxyphenoxyacetic acid	C_21_H_40_O_4_ C_10_H_12_O_5_	379.28 213.07	12.92 10.53	0.77 0.83	↓ ↓*		0.66 0.75	↓* ↓*
	7	Cis-gondoic acid	C_20_H_38_O_2_	328.32	11.86	0.61	↓*		0.58	↓*
	8	3-Hydroxydecanoyl carnitine	C_17_H_33_NO_5_	349.27	12.71	0.73	↓*		0.53	↓*
	9	Trans-2-hydroxycinnamic acid	C_9_H_8_O_3_	165.05	0.81	0.41	↓*		0.38	↓*
	10	Oxidized glutathione	C_20_H_32_N_6_O_12_S_2_	613.16	0.82	2.15	↑*		1.32	↑

↓ indicates a decrease; ↑ indicates an increase. ^*^P < 0.05

### ZSSG chemical ingredient analysis

**Fig. 5:**
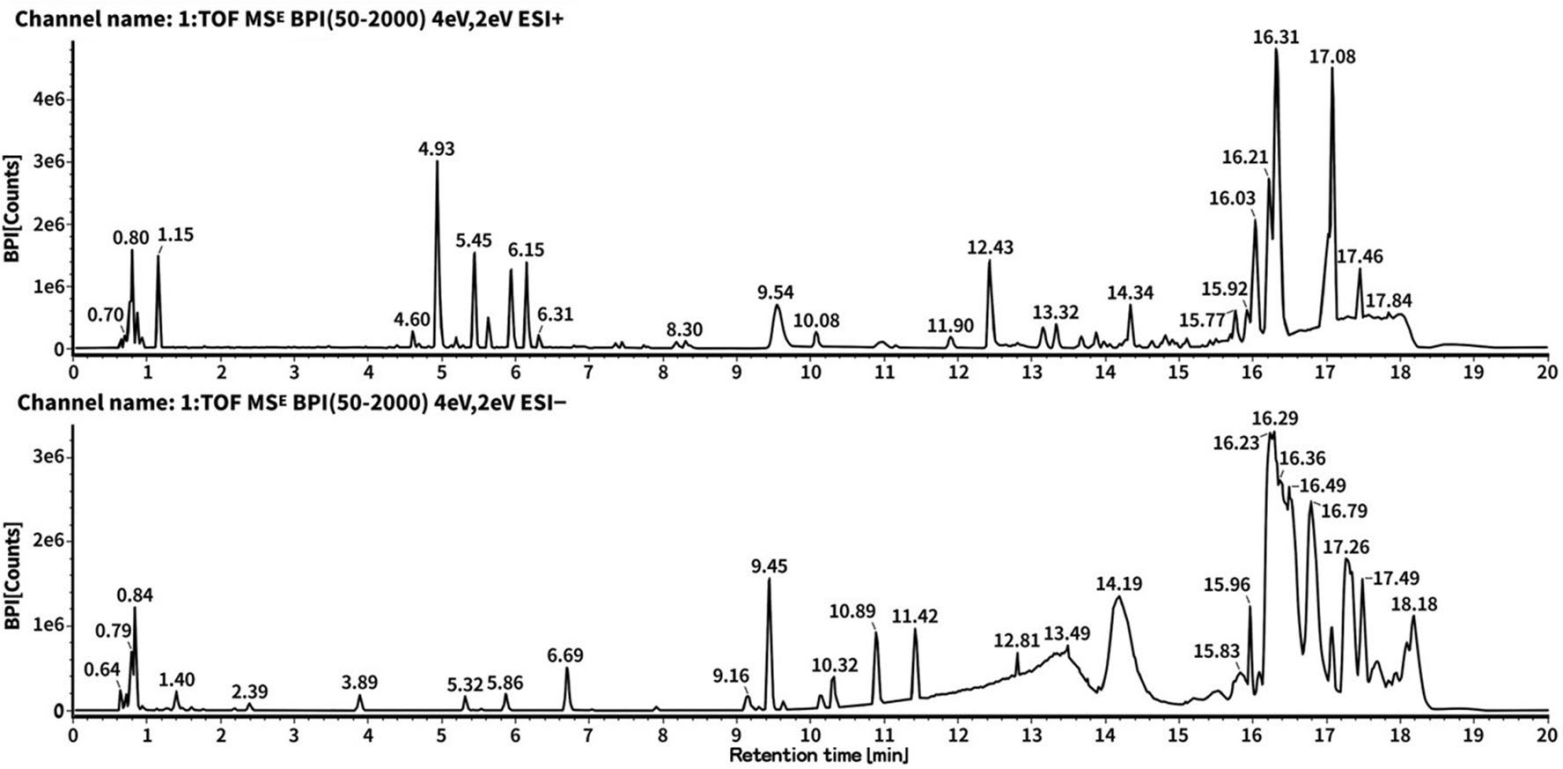
Chromatograms of ZSSG by UPLC-Q/TOF–MS analysis. Top graph: positive ion peak chromatograms of ZSSG; bottom graph: negative peak chromatograms of ZSSG

The chemical profile of ZSSG was analyzed by the chromatograms in the positive (ESI^+^) and negative (ESI^−^) modes (Fig. 5). All intensity peaks in the tested samples were considered. Chromatograms were obtained for ZSSG. 85 chemical ingredients were identified in ZSSG with various intensities by UPLC-QTOF-MS (Additional file 1: Table S1).

### ZSSG ingredient-target network analysis

**Fig. 6:**
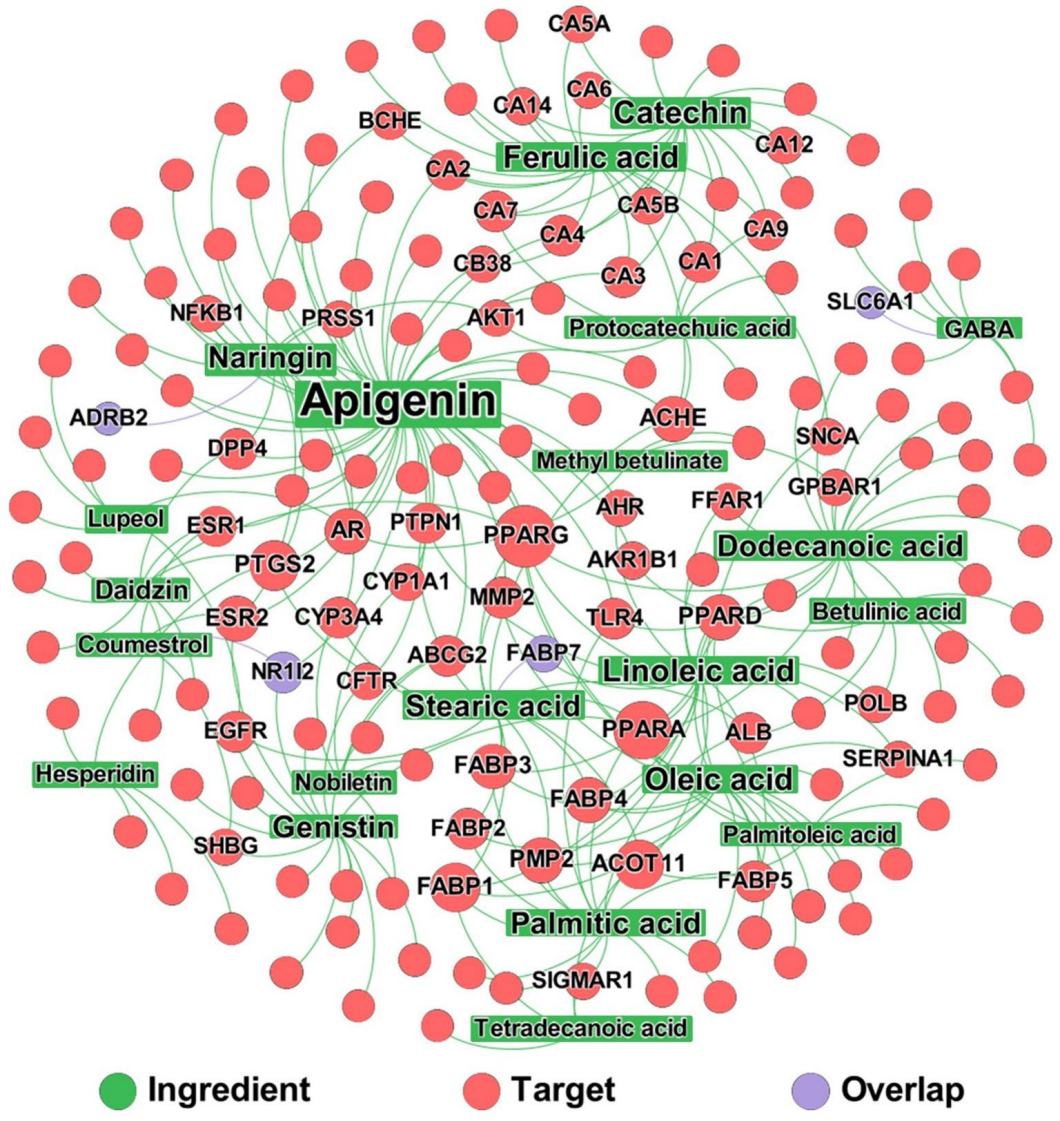
A bipartite ingredient-target network of ZSSG ingredients. The net contains 265 interactions connecting 21 ingredients (filled in green) and 161 protein targets (filled in red). The node size and label font size are proportional to the degree (connectivity). The label name is shown only when its degree is higher than 2. The purple nodes represent the shared targets by ingredient and insomnia

We started with 85 chemical ingredients identified by UPLC-Q/TOF–MS (Additional file 1: Table S1). We collected the protein targets for each ingredient of ZSSG from various databases and performed literature mining to establish an ingredient-target network. As shown in Fig. 6, the network contains 265 interactions connecting 21 ingredients and 161 protein targets. Interestingly, we found that 4 targets of ZSSG ingredients, ADRB2, FABP7, NR1I2, and SLC6A1, are also insomnia-associated proteins, which indicates that these four targets play important roles in the effective action of ZSSG against insomnia.

### Proximity between ingredient targets and insomnia genes in the interactome

The ingredients of ZSSG can be considered as drugs in that they bind to specific protein targets, affecting the indicated ability to carry out their function. Hence, we can use the network-based framework to predict the efficacy of drugs or polyphenols in indicated diseases [20, 30, 31] to also predict the effect of ingredients from herbal medicine. We obtained a final list of all identified ZSSG ingredients, for which 161 protein targets were collected. In total, 267 ingredient-target pairs were included in this study (Additional file 2: Table S2). 64 genes-associated with insomnia were collected from various databases and literature reviews (Additional file 3: Table S3). We found that 63 of 64 genes-associated with insomnia were mapped to the human interactome, consisting of 18,402 proteins and 485,412 interactions. We only considered the ingredients with at least two targets mapped in the interactome.

**Fig. 7:**
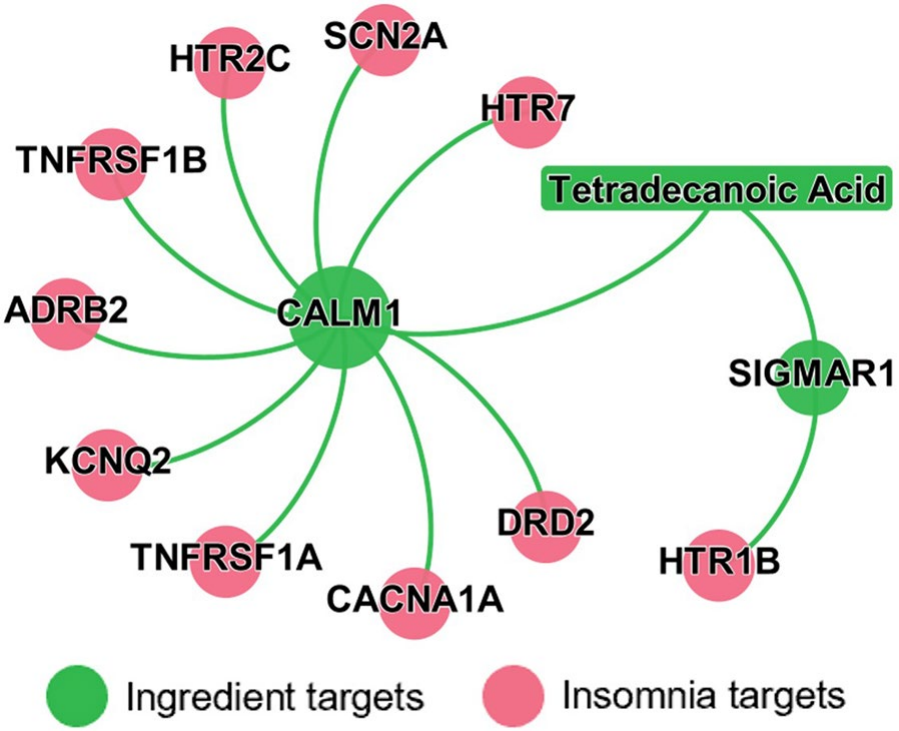
Network-based predicted mechanism of action for tetradecanoic acid in insomnia

Next, we calculated the network proximity between 63 genes-associated with insomnia and ingredient targets using the closest approach, finding that four target sets, each ingredient (four among twenty ingredients) targets and insomnia-associated genes, were significantly proximate to each other (Table 2, *Z*-score < -0.5, *P* ≤ 0.05). All the network proximity scores can be found in Additional file 4: Table S4. Tetradecanoic acid, also called myristic acid, is a common component of animal fats, vegetable oils, and dairy products. It is safe to consume less than 37.0 mg of tetradecanoic acid per day [32]. Previous studies have shown that tetradecanoic acid exhibits various pharmacological activities, such as antioxidant and anti-inflammatory activities in diabetic rats [33]. In the current study, we showcased the network-predicted candidate active ingredient tetradecanoic acid for insomnia. As shown in Fig. 7, tetradecanoic acid (*Z*-score = − 1.00) potentially affects several important proteins-associated with insomnia, including ADRB2 [34], DRD2 [35], and SCN2A [36]. Collectively, the network-based predicted tetradecanoic acid is a promising candidate active ingredient of ZSSG for insomnia treatment.

**Table 2:** The predicted therapeutic associations between ZSSG ingredients and insomnia

Ingredient	Structure	Mapped ingredient target protein (n)	Closest	*Z*-score	*P*-value
GABA		8	1.50	− 2.09	0.01
Stearic acid		16	1.63	− 1.68	0.02
Tetradecanoic acid		4	1.50	− 1.00	0.05
Coumestrol		4	1.25	− 0.95	0.05

Ingredients were ordered according to the network proximity (*Z*-score) of their targets to insomnia-associated genes, and ingredients with relatively larger *Z*-score were removed. References reported in the curated literature are shown.

### Network-based identification of potential ingredient combinations for insomnia

**Fig. 8:**
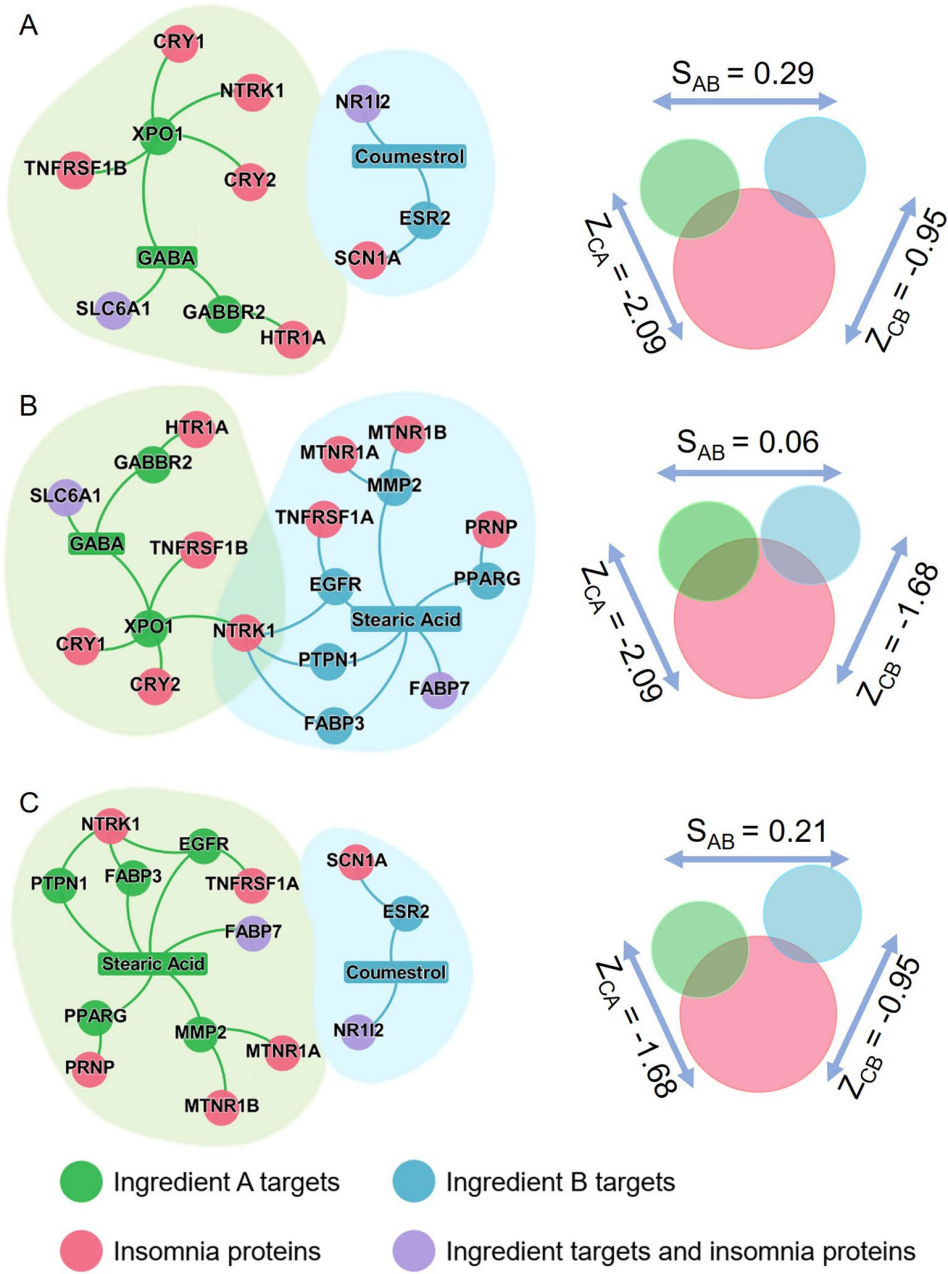
Network-based rational screening of chemical ingredient combinations for insomnia. The inferred mechanism-of-action networks for pairwise active ingredient combinations: (**A**) GABA plus coumestrol, (**B**) GABA plus stearic acid, and (**C**) stearic acid plus coumestrol. Z_CA_ and Z_CB_ indicate the network proximity (*Z*-score) between insomnia-associated proteins and ingredient A targets or ingredient B targets, respectively. S_AB_ indicates the separation score of targets between ingredient A and ingredient B

Drug combination therapy, aiming to reduce toxicity and increase therapeutic efficacy, plays a critical role in treating various cardiovascular diseases [37]. In the current study, we used a network-based method (complementary exposure) to identify efficacious ingredient combinations for insomnia. Among the four potential ingredients in Table 2, we found the following network-based predicted candidate ingredient combinations for insomnia: GABA plus coumestrol, GABA plus stearic acid, and coumestrol plus stearic acid (Fig. 8). It was noted that three protein targets, SLC6A1 of GABA, NR1I2 of coumestrol, and FABP7 of stearic acid, are also insomnia-associated proteins. The three chemical ingredients and their corresponding potential targets may be involved in the major mechanism of action of ZSSG in controlling insomnia. All the predicted combinations can be found in Additional file 5: Table S5.

### Identification of the selective ingredients in mouse brains

**Fig. 9:**
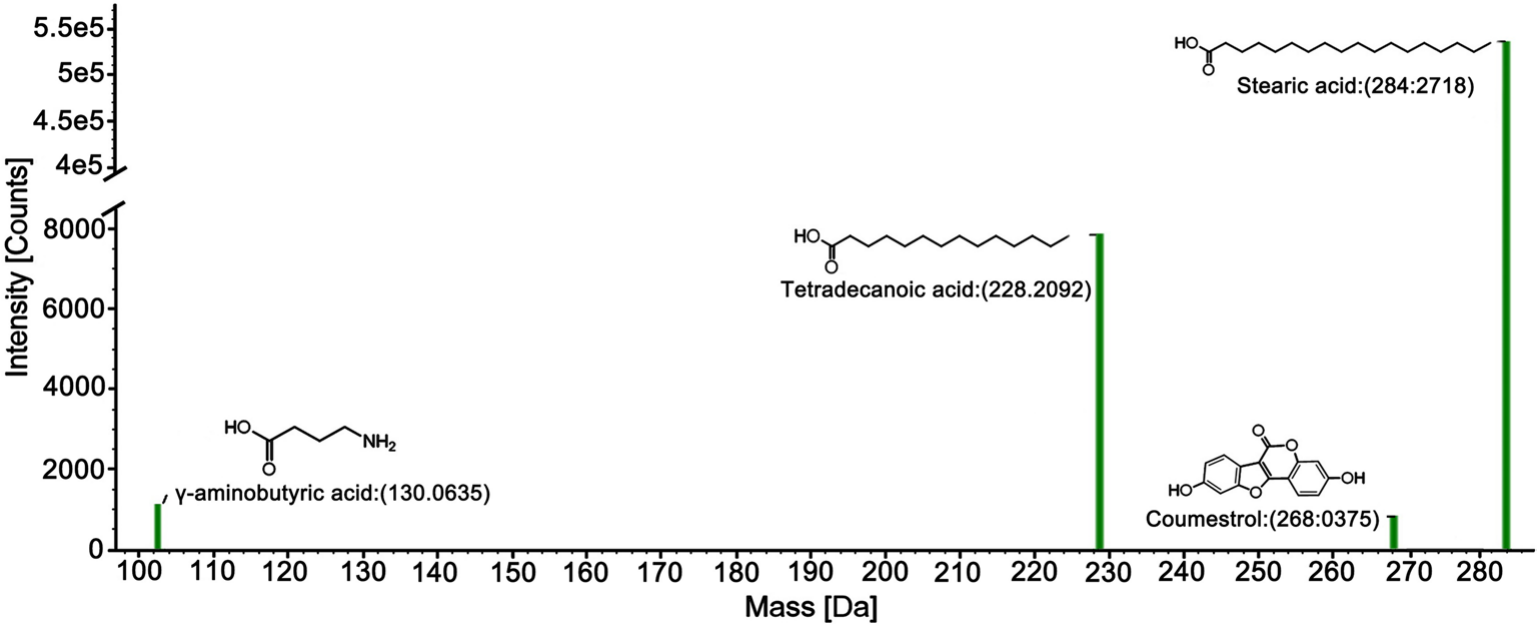
Identification of the 4 prototype ingredients in mouse brains by UPLC-Q/TOF–MS/MS. Mass spectra of each peak were obtained and used for compound identification. The accurate masses and fragment ions of each peak were confirmed by database searching

In accordance with the predictive active ingredients by network-based approach, we identified the indicated ingredients in mouse brains using high-resolution and sensitivity UPLC-Q/TOF–MS/MS system. The error results indicated the precision and reliability were less than 5 ppm. As anticipated, the 4 key ingredients GABA, stearic acid, tetradecanoic acid and coumestrol were identified in mouse brain tissues, as shown in Fig. 9. The 4 identified prototype ingredients provide a basis for studying their hypnotic effects.

## Discussion

The present study investigated the effect of ZSSG on sleep in mice. Our findings revealed that ZSSG had no direct effect on sleep in normal mice. However, in sodium pentobarbital-induced sleeping mouse model, ZSSG significantly improved sleep by decreasing sleep latency, prolonging sleep duration, and increasing brain GABA content. Similarly, in sodium barbital-induced sleep in mice, ZSSG decreased sleep latency. According to the criteria for improving sleep in the Technical Standards for the Testing and Assessment of Health Food in China, ZSSG exhibits an effect on improving sleep. Mechanistically, ZSSG may suppress central nervous excitability by increasing the brain content of GABA to improve sleep quality in mice. Furthermore, ingredient combinations, including GABA and coumestrol, may have a synergistic effect on sleep through the regulation of the corresponding subnetwork. Additionally, new metabolites of ZSSG may have positive effects on sleep in mice. Lastly, the use of proximity network medicine shows promise in exploring the mechanism of action of herbal medicine, and the online platform we have developed holds significant ramifications for researchers in the field of TCM. The platform provides a user-friendly and cost-free tool for conducting network proximity between a set of herbal active ingredients’ targets and a set of disease (symptom) related genes. These calculations typically require specialized expertise and resources, but our platform offers a convenient solution, thereby enabling a wider range of researchers to leverage the benefits of network medicine in their research, regardless of their technical proficiency.

Some scholars in an early study found that spinosyn (10 or 15 mg/kg), a flavonoid isolated from ZSS, enhanced pentobarbital-induced sleep in mice and rats, characterized by reduced sleep latency and increased sleep duration, probably via the serotonergic system and postsynaptic 5-HT1A receptor [38, 39]. Until now, the pharmacological investigation of ZSS for the treatment of insomnia has mainly focused on rat models. For instance, extensive studies from para-chlorophenylalanine-induced insomnia model rats demonstrated that ZSS aqueous extract (30 g/kg) or ZSS alcohol extract (403.38 mg/kg) significantly enhanced the serum concentration of 5-TH and enhanced sleeping behaviors, indicating that the extract was effective in treating insomnia in rats [40, 41]. Another main ingredient GABA, one of the major inhibitory neurotransmitters in the nervous system, generates a neuroinhibitory effect by binding with its receptor. It was reported that activation of the GABA receptor is beneficial for sleep [42]. In the current study, after treatment for 30 days, ZSSG at a dose of 0.57 g/kg (including 0.25 g/kg GABA) improved sleep quality in sodium pentobarbital- and sodium barbital-treated male Balb/c mice. None of the studies showed any toxicities or side effects.

In this study, owing to constraints related to the number of animals and the duration of the experiment, conducting sleep tests on all nine groups—including control, model, positive, low and high dose ZSS, low and high dose GABA, and low and high dose ZSSG—is not feasible within the scope of this study. The challenge lies in accurately observing the designated time for all 90 animals within the relatively brief span of a batch. Consequently, we opted for metabolic analysis instead of sleep tests. By comparing metabolic differences between ZSSG, ZSS or GABA alone, we could indirectly infer the advantages of ZSSG in enhancing sleep regulation. Previous studies have indicated that the metabolic process is vital in the regulation of sleep–wake cycles [43, 44]. Our findings suggest that 10 metabolites demonstrated significant differences in the ZSSG group in comparison to single-agent (ZSS or GABA) group. For instance, previous studies have shown that mice administered ginseng glycoprotein exhibited increased levels in brain tissue and promoting sleep quality [45]. Moreover, uridine, a sleep-promoting substance derived from the brainstem of sleep-deprived rats, resulted in increased sleep frequency upon intravenous injection [46, 47]. Based on our results and the current literature, it is reasonable to speculate that ZSSG may have an advantage in regulating metabolism and improving sleep quality compared to single-agent formulas (ZSS or GABA).

ZSSG, a mixture of ZSS and GABA, has many components and complex pharmacological mechanisms of action and therefore may have the therapeutic characteristics of multi-target and multi-pathway regulation. To investigate the systemic regulatory mechanism of how ZSSG confers its sleep-improving effect, we used a network-based in silico methodology to characterize the relationship between ZSSG ingredients and insomnia. Network-based framework methodology, measuring the relative proximity between the disease-associated genes and drug targets in the human PPI network, has been successfully used to analyze the relationship between diseases and drugs [20, 30]. Interestingly, we found that four potential active ingredients in ZSSG and the corresponding targets may be involved in controlling sleep.

As expected, our study found that ZSSG significantly increased the brain content of GABA in mice. It was reported that activation of GABA neurons in the ventral tegmental area elicited sleep, while lesioning these neurons resulted in increased wakefulness for at least 4 months [48]. In line with this study, our results demonstrated that GABA is top 1 ranked by the proximity network (*Z*-score: -2.09), which indicated that GABA is the most potent active component in ZSSG in controlling sleep. This result provided further support for the reliability of the proximity network. Solute carrier family 6 member 1 (SLC6A1) encodes GABA transporter protein type 1 (GAT1), which is the most abundant GABA transporter in the brain. A previous study showed that GAT1 knock-out mice spent a longer time in rapid eye movement (REM) sleep than wild-type mice, which indicates that GAT1 plays a critical role in controlling sleep [49]. It was noted that the target of GABA, SLC6A1, is also a protein-associated with insomnia. Based on our results combined with the indicated literature, we conclude that ZSSG may suppress central nervous excitability, at least in part, by regulating the levels of GABA to improve sleep quality in mice.

With the network-based ingredient screening model, we highlighted four major ingredients (GABA, stearic acid, tetradecanoic acid, and coumestrol) of ZSSG that might be able to regulate sleep quality. Taking stearic acid as an example, experimental research has demonstrated that this ingredient binds to FBAP7 [50]. FBAP7 knock-out mice displayed an increase in wake during the light phase, indicating that FBAP7 is required for normal sleep inhibition [51]. More recently, it was reported that a reduction in stearic acid was observed in stool samples from patients with obstructive sleep apnea disease [52], which indicates that stearic acid may play an important role in controlling sleep. In particular, one additional ZSSG ingredient examined in the current study is pregnane xenobiotic receptor (PXR, NR1I2). NR1I2 knock-out mice displayed abnormal theta rhythm during sleep [53]. Coumestrol, a member of the isoflavonoid family, is a natural product with an estrogen-like structure. Coumestrol was reported to act as a human NR1I2 competitive antagonist [54]. It was noted that the target of coumestrol, NR1I2, is also insomnia-associated with the corresponding gene. Hence, to some extent, this experimental evidence supports the network-based predictions of the current study.

In addition to the roles of a single potential active ingredient, three pairwise combination ingredients (GABA plus coumestrol, GABA plus stearic acid, and coumestrol plus stearic acid), which may have synergistic effects, may be another mechanism for the improvement of sleep by ZSSG. It was reported that the network-based strategy can be successfully used to screen drug combinations for potentially new indications [21, 28]. Accordingly, we ranked all possible ingredient pairs by separation score ${S}_{AB}$, and then restricted these ingredient pairs with “Complementary Exposure” pattern to insomnia disease module. We found three network-based predicted candidate ingredient combinations for insomnia. Among the three pairs of ingredient combinations, GABA plus coumestrol or stearic acid may have synergistic effects on insomnia. This may be the reason why ZSS plus GABA as a food formulation mediates sleep quality.

The current study had several limitations. First, due to the lack of available corresponding data, the current existing relevant background information cannot guarantee that all the targets of ZSSG ingredients and the insomnia-related genes are collected, and the results cannot confirm the whole network framework of insomnia and each ingredient. The incomplete human interactome may also affect the accuracy of prediction. Second, many ingredients of ZSSG were combined to exert a sleep-improving effect. However, the network medicine framework methodology was constrained by the current algorithm and other background knowledge. Although the combination of multiple ingredients is important in the field of herbal research, a method to calculate the combined effects of more than two ingredients is currently unavailable.This is another limitation and deserves further investigation. Third, although experimental data (the association between GABA and sleep) have validated some of the predicted positive ingredients, further experiments are necessary to validate the predicted sleep-improving effects and the corresponding molecular subnetwork.

## Conclusions

This study demonstrated that ZSSG possesses a significant effect in the hypnosis experiment, with the best dose being 0.57 g/kg. These findings provide both theoretical and experimental evidence for the clinical application of ZSSG as sleep-enhancing health food. Moreover, metabolic analysis revealed that ZSSG has the advantages in regulating the metabolic pattern-associated with sleep compared with the single-agent ZSS or GABA. Additionally, the network medicine framework-based approach proposed in the current study shows promise in the identification of potential active ingredients and the corresponding molecular network mechanism of action on sleep-improving effects. Furthermore, our results also support that the potential four active ingredients and three pairwise ingredient combinations could serve as quality markers-associated with insomnia. In combination with experimental validation, this network framework could serve as a useful method to enhance the efficiency of exploring the active ingredients and mechanisms of herbal medicines.

### Supplementary Information

**Additional file 1: Table S1.** A list of 85 ingredients from ZSSG.

**Additional file 2: Table S2.** List of ingredient targets.

**Additional file 3: Table S3.** Insomnia-associated genes.

**Additional file 4: Table S4.** Network proximity score.

**Additional file 5: Table S5.** SAB score.

## Data Availability

The datasets and all the relevant codes are available from the corresponding author.

## Abbreviations

ZSS: Ziziphi Spinosae Semen
GABA: γ-Aminobutyric acid
ZSSG: ZSS and GABA
UPLC: Ultra-performance liquid chromatography
Q/TOF: Quadrupole time of flight
MS: Mass spectrometry
Pent: Sodium pentobarbital
Barb: Sodium barbital
Diaz: Diazepam
